# Critical appraisal of perioperative neuroprotection evidence in aortic arch surgery: a correspondence

**DOI:** 10.1097/MS9.0000000000004470

**Published:** 2025-12-03

**Authors:** Gaaitri Lohano, Maryam Waqar, Pari Kotak, Govinda Lohano, Hermann Yokolo

**Affiliations:** a Department of Internal Medicine, Liaquat University of Medical and Health Sciences, Jamshoro, Pakistan; b Department of Internal Medicine, DOW Medical College, Karachi, Pakistan; c Department of Research, Medical Research Circle (MedReC), Goma, DR Congo

**Keywords:** aortic arch surgery, critical appraisal, perioperative neuroprotection

*To the Editor,*

We have carefully reviewed the meta-analysis titled “Neuroprotection in Aortic Arch Surgery: A Meta-Analysis of Hypothermia and Selective Cerebral Perfusion on Perioperative Stroke and Cognitive Outcomes” by Patel *et al* published in the Annals of Medicine and Surgery^[1]^. The authors present a scientifically relevant and timely contribution that enhances the current understanding of neuroprotective strategies in aortic arch surgery. Their analysis provides valuable insights into perioperative neurological and cognitive outcomes, offering an evidence base that warrants further consideration in both clinical decision-making and future research. This article complies with the TITAN 2025 guidelines – repressing the reporting and use of AI^[2]^.

First, there is an absence of standardized neurological outcome assessment. Although the paper reports “perioperative stroke,” it never specifies whether outcomes were clinically or radiologically confirmed, nor does it detail the use of validated neurocognitive scales. This lack of consistency across studies allows misclassification bias, weakening the reliability of results and limiting their translation into perioperative neuromonitoring protocols^[3]^.

Second, all reported endpoints are confined to the immediate postoperative period, with no follow-up beyond hospitalization. Without long-term data, late cognitive decline or delayed embolic events cannot be excluded. This concern is reinforced by the GERAADA long-term registry, which reported that approximately one-quarter of patients developed secondary neurological deficits up to a decade after surgery, indicating that early perioperative findings do not ensure durable neuroprotection. Clinicians, therefore, cannot infer sustained neuroprotection from these findings, which substantially diminishes the clinical weight of the authors’ conclusions^[4]^.

Third, despite providing strong evidence for hypothermia, studies on neuroprotection strategies do not indicate how cerebral perfusion flow is affected by temperature. During hypothermic circulatory arrest, cerebral protection depends not only on the individual’s body temperature but also on how much blood flows through the brain during perfusion to reach the affected area. The connection between temperature and perfusion flow, as shown by Lucy *et al*, is complex, leading to changes in oxygen metabolism in the brain, suggesting that neuroprotection cannot be solely indicated by temperature^[5]^. It is essential to keep evaluating both hemodynamic and metabolic parameters to ensure cerebral protection across various temperature conditions. Therefore, future studies should include assessment of perfusion flow along with temperature monitoring to more accurately determine neuroprotective measures.

Lastly, this article demonstrates worthwhile insights into the neurocognitive outcomes of different degrees of hypothermia during aortic arch surgery, but it does not touch on causal factors that can lead to variance in the findings, as suggested by the flouted differences in structured verbal memory and brain connectivity^[6]^. These studies suggest the need to conduct more research in order to streamline brain perfusion and neuroprotection measures during hypothermic circulatory arrest.

Overall, while Patel *et al* offer valuable insights into neuroprotection during aortic arch surgery, methodological inconsistencies and limited follow-up reduce clinical applicability, underscoring the need for standardized assessments and long-term evaluation in future research.

## Data Availability

Not applicable.
